# Clinical profile of microsporidial keratoconjunctivitis in healthy individuals of China —new species and neglected risk factors

**DOI:** 10.1186/s12348-026-00596-9

**Published:** 2026-05-22

**Authors:** Yingnan Xu, Xuguang Sun, Shanshan Xu, Shijing Deng, Yang Zhang

**Affiliations:** 1 The Department of Ophthalmology, The Affiliated Eye Hospital of Nanjing Medical University, 138 Han-Zhong Road, Nanjing, 210029 China; 2 Beijing Ophthalmology & Visual Sciences Key Lab, Beijing Tongren Eye Center, Beijing Institute of Ophthalmology, Beijing Tongren Hospital, Capital Medical University, Beijing, 100005 China; 3 Beijing Ophthalmology & Visual Sciences Key Lab, Department of Corneal Diseases, Beijing Tongren Eye Center, Beijing Tongren Hospital, Capital Medical University, Beijing, 100005 China

**Keywords:** Keratoconjunctivitis, Infectious, *Enterocytozoon bieneusi*, *Encephalitozoon hellem*, Contact lenses, Parrots

## Abstract

**Objective:**

To characterize microsporidial keratoconjunctivitis (MKC) in immunocompetent individuals in Mainland China, including novel etiologies and risk factors.

**Methods:**

A prospective analysis of 20 MKC patients in 2025, including clinical features, pathogens (via corneal scrapings and metagenomic sequencing), risk factors and etc.

**Results:**

All patients were misdiagnosed for a median of 1 month. Patients (mean age 28.5 years, 13 F) showed Encephalitozoon hellem (65.0%), E. bieneusi (15.0%, first reported in MKC), and Vittaforma corneae (15.0%). Key risks included bird contact (70.0%, mostly psittacines), contact lens use (40.0%), and water exposure (15.0%). The most common symptom was redness (85.0%); limbal fluorescein positivity occurred in 65.0%. Topical 0.02% PHMB cured 90.0% of 20 cases; one recurrence followed treatment stop. Some E. hellem cases linked to parrots showed potential zoonotic transmission.

**Conclusion:**

MKC in China involves E. bieneusi and parrot-associated E. hellem. Limbal staining aids diagnosis; PHMB is effective. Zoonotic risks related to Psittacine birds and contact lens use require clinical attention.

## Introduction

Microsporidia include a group of unicellular spore-forming obligate eukaryotic parasites of invertebrates and vertebrates [1]. Ocular infection is the second most common manifestation of human microsporidiosis [1, 2]. Since first report in early 2000s, *Encephalitozoon* spp. and *Nosema* spp. were reported in some Asian regions to cause ocular surface infection of immunocompetent individuals, named microsporidial keratoconjunctivitis (MKC) [3, 4]. However, only a small case series of MKC were published by our group in Mainland China [5]. Besides, diagnostic delay is a key diagnostic bottleneck of microsporidial keratoconjunctivitis and other ocular infections, which is highly consistent with the diagnostic difficulties of fungal keratitis [6]. Misdiagnosis time differed among studies, ranging from a few days to several months [7, 8]. An elevated misdiagnosis rate may directly compromise patient prognosis. Corneal scraping, molecular diagnostics, in vivo confocal microscopy (IVCM) and anterior segment Optical Coherence Tomography have been applied to the diagnosis of MKC [9–11]. In order to explore the clinical and etiological characteristics of microsporidium infection in mainland China in-depth, we prospectively collected cases of MKC consecutively.

## Materials and methods

We prospective collected consecutive patients with unilateral or bilateral keratoconjunctivitis diagnosed as MKC in Eye Hospital of Nanjing Medical University, from January 2025 to December 2025. This study was approved by an ethical committee of Eye Hospital of Nanjing Medical University (Ethical Review Number: 2024009), and adhere to the principles of the Declaration of Helsinki. The basis of diagnosis were positive results of corneal epithelial scrapings and/or metagenomic next-generation sequencing (mNGS). Informed consent was acquired from all patients. The demographic data, clinical history, risk factors, visual acuity, and slit-lamp biomicroscopic examination results of 20 patients were collected in detail.

### Immunological examination

Blood samples were collected from all patients. Complete blood count, lymphocyte subsets, immunoglobulins, complements and autoantibodies were conducted. Enzyme-Linked Immunosorbent Assay (ELISA) for HIV types I and II was performed. Meanwhile, a comprehensive clinical examination was carried out by internists to rule out any other signs of immunosuppression, including history of immunosuppressive drug use, history of administration of immunoglobulin and other immune-related blood products, history of malignant tumors and relevant treatments, and history of autoimmune diseases.

### Corneal scrapings and etiological examination

Corneal scrapings and culture were conducted in all consented patients to evaluate microsporidia and other pathogens. After instillation of a drop of topical anesthetic agent (0.5% proparacaine hydrochloride), coarse epithelium was scraped by a sterile spatula under microscopy. Removed epithelium was smeared onto two glass slides for microscopic examination firstly, and was inoculated onto various media for bacterial, fungal and acanthamoeba culture immediately. Other smear of cornea and conjunctival sac were tested for adenoviruses and human herpes virus 1–5. Based on our former study [12], all scrapings were examined after sequentially staining with Giemsa stain and 0.1% calcofluor white (CFW) stain. Some cases with larger number of scraped cells and typical spores, were also stained with Gram stain and modified Ziehl-Neelsen stain/1% H_2_SO_4_ (Kinyoun stain) for comparison with Giemsa and CFW stain.

### Clinical next-generation metagenomic sequencing (mNGS)

Removed epithelium were collected into DNA/RNA-Shield and stored at -20℃ temporarily. DNA were extracted and purified from sample by QIAamp DNA Micro Kit (QIAGEN, Hilden, Germany). The DNA library were constructed by QIAseq™ Ultralow Input Library Kit (QIAGEN, Hilden, Germany), and sequenced on the NovaSeq6000 instrument (Illumina, San Diego, USA). Under quality control, raw sequencing data were cleaned without human reads by using SNAP software.

Reads were filtered to retain sequences with a minimum length of 50 bp, a Q30 ratio ≥ 85% [13]. Remaining sequence were aligned to the Microbial Genome Databases (https://ftp.ncbi.nlm.nih.gov/genomes/). As obligate intracellular pathogens, microsporidia evade full nucleic acid release and exhibit low extraction yield, leading to possibly fewer mNGS reads. Any microsporidia supported by at least 3 specific non-overlapping mNGS reads was classified as a positive result [14]. Organisms detected in NTCs were routinely eliminated to exclude background interference; however, species with a sample-to-NTC read ratio exceeding 10-fold were not excluded. Contaminants were filtered by comparing against negative control data and removing taxa enriched in reagents.

### Statistical analysis

All statistical analyses were performed with SPSS 26 (IBM Corp., Armonk, NY, USA). Significance level α = 0.05; *P* < 0.05 was considered statistically significant.

Means, median, standard deviations, and ratio data were calculated. Decimal Best corrected visual acuity (BCVA) of each affected eye before and post treatment was compared by the Wilcoxon paired signed-rank test. Number of plaque-like epithelial lesion were graded into 1–5, and number of anterior stromal infiltration were graded into 1–4. Higher grades indicated more severe epithelial lesions or stromal infiltration. Decimal BCVA was converted to logMAR BCVA. Higher logMAR values represented worse visual acuity. Spearman’s rank correlation was used to analyze associations between lesion/infiltration grades and before-/post-treatment logMAR BCVA.

## Results

This case series prospectively collected 20 cases of MKC (13 Females, 7 males). All patients were healthy with negative serological tests of HIV. Their average age was 28.5 ± 22.7 yrs old (range from 5 to 69 yrs old). Twelve cases were bilateral: four with simultaneous onset, eight with sequential onset. Amongst unilateral cases, right eye was involved in 6 cases and left eye in 2 cases. Duration of symptom before confirmed diagnosis ranged from 0.5 to 24 months (median 1 month). Three main risk factors of MKC was contacting possible contaminated water (15.0%, 3/20), contact lens (including orthokeratology lens) wearing (25.0%, 5/20), and contacting birds (70.0%, 14/20, including 13 cases related to Psittacine birds). Main infected species of microsporidia included *Encephalitozoon hellem* (65.0%, 13/20), *E. bieneusi* (15.0%, 3/20), *Vittaforma corneae* (15.0%, 3/20). All cases were negative for bacterial, fungal, acanthamoeba, adenoviruses and human herpes virus 1–5.

Redness (85.0%, 17/20), decrease in vision (35.0%, 7/20), foreign-body sensation (35.0%, 7/20) and watering (35.0%, 7/20) were four major symptoms. The number of plaque-like epithelial lesion were less than 20 in 65.0% of cases (13/20). Peripheral or central and peripheral sub-epithelial infiltration accounted for 60.0% (12/20). An interesting sign of MKC was positive fluorescein staining of limbus in 65.0% (13/20) of cases. While positive fluorescein staining of bulbar conjunctiva were only found in 30.0% (6/20) of cases.

All cases were misdiagnosed at the initial presentation. Adenovirus keratoconjunctivitis (14/20), dry eye (3/20), allergic conjunctivitis (3/20) and Thygeson superficial punctate keratitis (TSPK) (3/20) were four major misdiagnoses. 0.02% Polyhexamethylene biguanide hydrochloride (PHMB) (17/20), cyclosporin eye drops (6/20) and oral albendazole (6/20) were three main treatment in this case series. Currently, 95.0% (19/20) of patients were cured without recurrence within 2–16 months of follow-up. One case of recurrence started after drug withdrawal at 3 months.

**Fig. 1:**
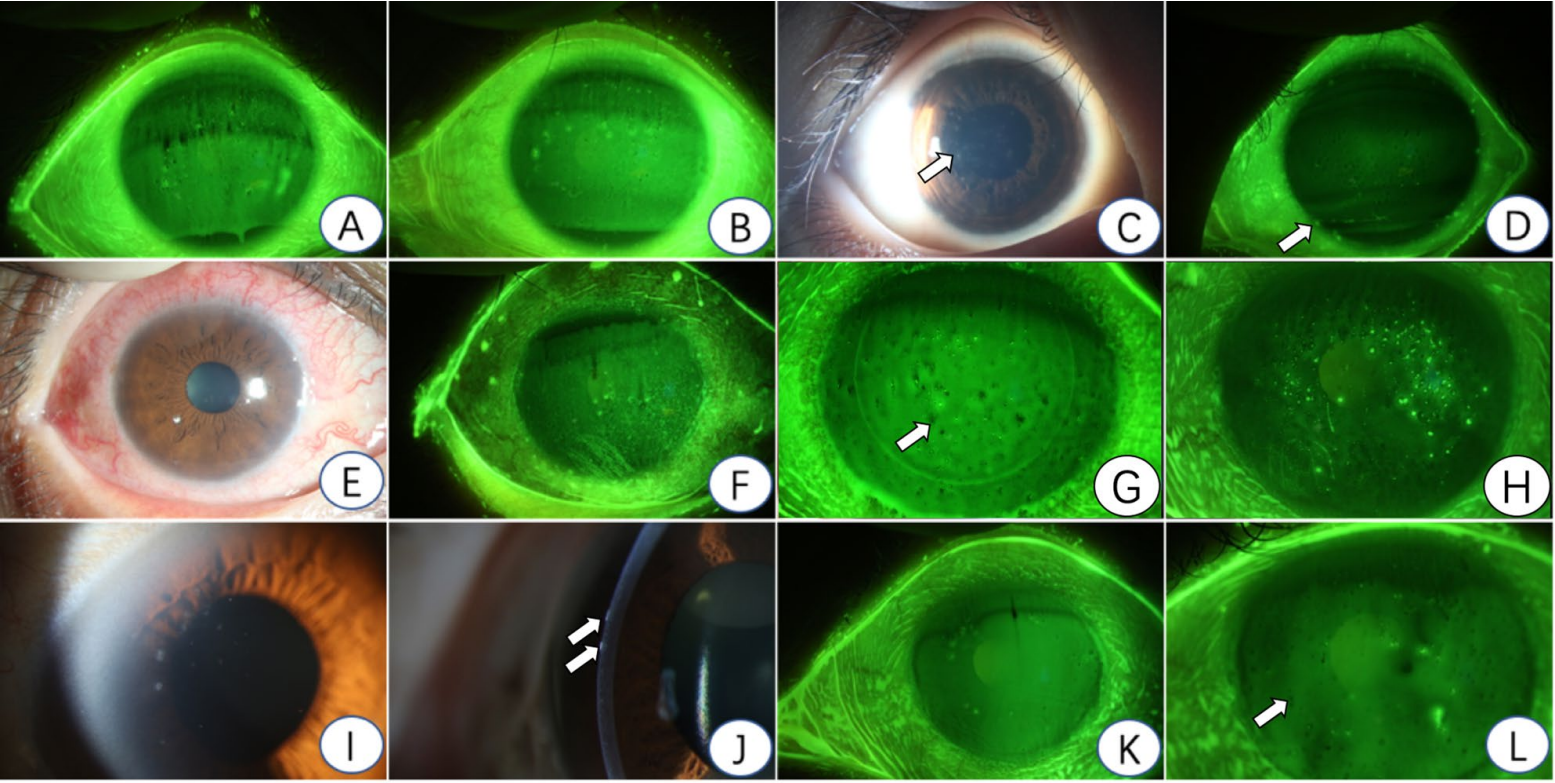
Slit-lamp photographs of typical signs. **A**-**B**: Case 2, x10; **C**-**D**: Case 5, rough corneal epithelium and subepithelial infiltration (C, arrow), limbal epithelial lesions with positive fluorescein staining (D, arrow) x10; **E**-**F**: Case 6, x10; **G**: Case 10, counterstaining epithelial lesion (arrow), x16; **H**: Case 11, x16; **I**-**K**: Case 15 (first visit), Plaque-like punctate white elevated lesions (J, arrow), I-J, x25, K, x10; **L**: Case 15 (3 days after the first visit), Plaque-like epithelial lesion increased and location changed, counterstaining epithelial lesion became obvious (arrow), x16

**Fig. 2:**
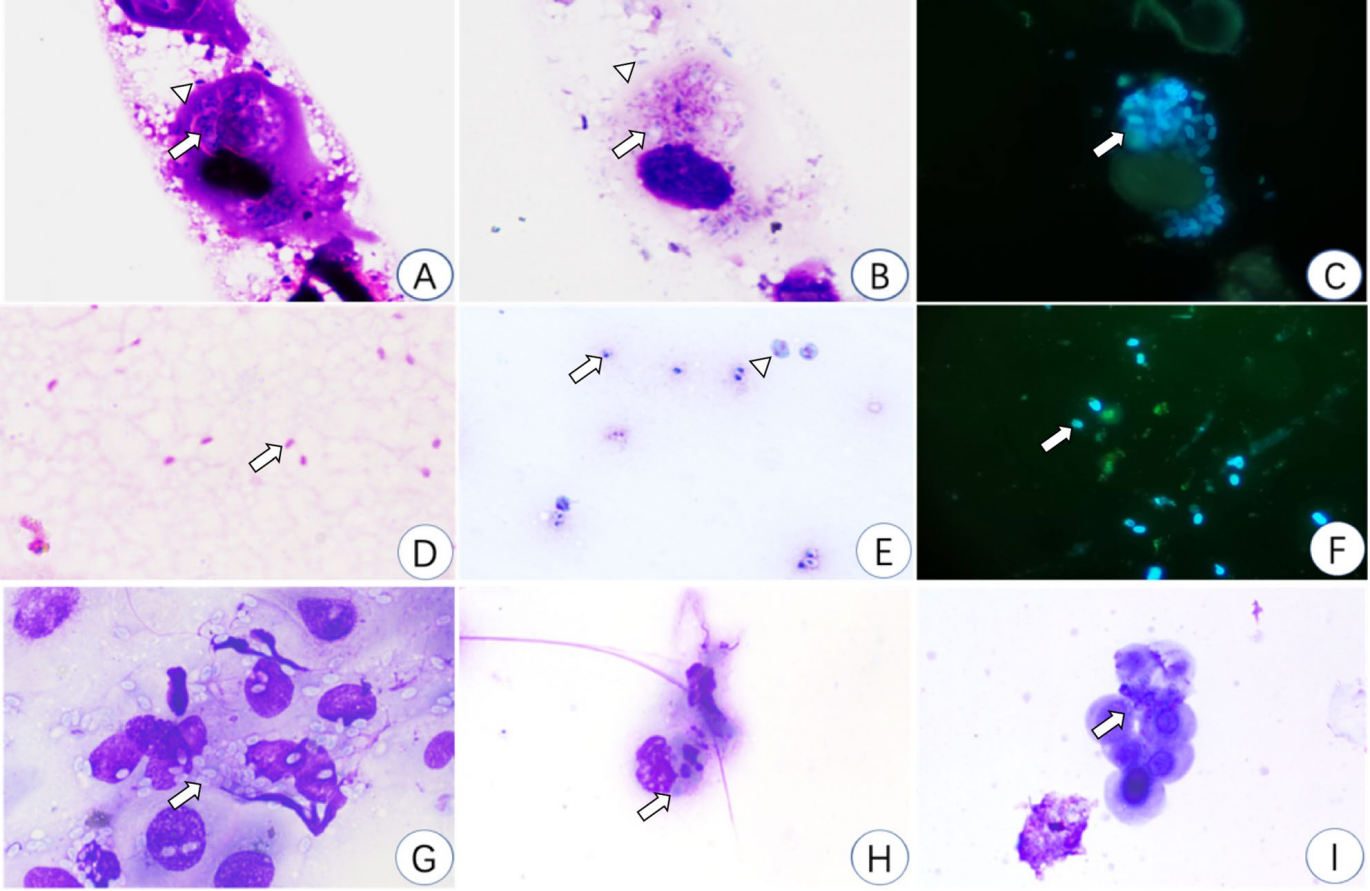
Microscopic photographs of corneal scraping under different staining, 1 000x. **A**-**C**. intracellular (arrow) and extracellular (arrowhead) microsporidal spores of Case 20 of same visual field, A: Gram staining, B: Giemsa staining; C. CFW staining; **D**-**F**. Case 4; D: acid-fast, oval microsporidial spores (arrow) in modified Ziehl-Neelsen stain/1% H2SO4 (Kinyoun stain); E. Giemsa staining demonstrated spores(arrow) and meronts (arrowhead). F. CFW staining only showed spores (arrow); **G**: spores (arrow) on Giemsa staining of Case 15; **H**, **I**: Giemsa staining of Case 19 and Case 2., Spores inside the epithelial cell were relatively transparent (arrow)

Demographic and clinical features see Tables 1, 2, 3. Five typical cases are described in details. Slit-lamp photographs of typical signs see Fig. 1. Typical microscopic photographs of corneal scraping see Fig. 2.

**Table 1:** Demographic and clinical features of 20 cases of MKC

Case NO.	Gender	Age	Possible risk factors	Duration of symptom before confirmed diagnosis	Corneal scrapings			mNGS		
					Giemsa	Calcofluor White		species	NO. of sequences	
1	F	10	orthokeratology lens wearing, contacting outdoor stream	2 months	-	+		patient refused		-
2	M	25	wearing contact lens	2 months	+	-		*Enterocytozoon bieneusi*		18
3	M	8	Unknown	6 months	Patient refused			*Enterocytozoon bieneusi*		653
4	F	37	contacting Psittacine birds	3 months	+	+		*Encephalitozoon hellem*		314
5	F	5	contacting Psittacine birds	2 months	-	-		*Encephalitozoon hellem*		42
6	F	45	contacting outdoor spring water	1 month	+	-		*Enterocytozoon bieneusi*		34
7	M	13	contacting Psittacine birds, orthokeratology lens wearing	0.7 month	+	+		*Encephalitozoon hellem*		**31**
8	M	48	contacting Psittacine birds	1 month	+	+		*Encephalitozoon hellem*		**34**
9	F	61	contacting Psittacine birdsx	2 months	+	+		*Encephalitozoon hellem*		23
10	M	19	contacting Psittacine birdsx	0.5 month	+	+		*Encephalitozoon hellem*		402
11	F	66	contacting Psittacine birdsx	0.5 month	+	+		*Encephalitozoon hellem*		70
12	M	23	contacting Psittacine birdsx	0.8 month	+	+		*Encephalitozoon hellem*		144
13	F	14	orthokeratology lens wearing; contacting Psittacine birdsx	1 month	+	+		*Encephalitozoon hellem*		22
14	F	12	contacting Psittacine birdsx	0.8 month	+	+		*Encephalitozoon hellem*		25
15	F	69	contacting Psittacine birds	0.5 month	+	+		*Vittaforma corneae*		5576
16	F	15	orthokeratology lens wearing; contacting Psittacine birds	0.8 month	+	+		*Encephalitozoon hellem*		3*
17	F	43	contacting Psittacine birds	1 month	+		+	*Encephalitozoon hellem*		3*
18	M	22	Swimming in sea, touching pigeons	1 month	+	+		*Vittaforma corneae*		10
19	F	27	traveling to Inner Mongolia, the specific reason is unknown.	24 months	+	-		*Vittaforma corneae*		11
20	F	7	contacting Psittacine birds	1 month	+	+		*Encephalitozoon hellem*		5*

*As obligate intracellular pathogens, microsporidia tend to yield fewer mNGS reads; Microsporidia with no fewer than 3 specific non-overlapping mNGS reads were defined as positive, with no corresponding sequences detected in NTCs. Corneal scrapings were also positive

**Table 2:** Symptoms and signs of 20 Cases of MKC

Case NO.	Symptoms	Signs							
		coarse, multifocal, punctate, raised plaque-like epithelial lesion			anterior stromal infiltration		other signs on ocular surface		
		number	position	fluorescein staining pattern*	number	position	palpebral conjunctiva	Bulbar conjunctiva	limbus
1	redness, secretion	< 10	peripheral	A	10 ~ 20	central and peripheral	congestion, follicle	congestion	F+**
2	redness, foreign-body sensation	< 10	central and peripheral	A + C	< 10	peripheral	none	none	F-
3	watering, foreign-body sensation	10 ~ 20	central	D	< 10	central	none	none	F-
4	redness, pain, decrease in vision	10 ~ 20	central and peripheral	A + B	10 ~ 20	central and peripheral	congestion	congestion, F+	F+
5	redness, pain, decrease in vision	21–40	central and peripheral	D	21–40	central and peripheral	congestion, follicle	congestion, F+	F+
6	redness, foreign-body sensation	21–40	central	A + B	< 10	central	congestion, follicle	congestion, chemosis, F+	F+
7	redness, foreign-body sensation	< 10	peripheral	A	none	none	congestion	congestion, F+	F+
8	redness, itching, secretion	10 ~ 20	central and peripheral	A + C	none	none	none	congestion	F-
9	redness, pain, watering, foreign-body sensation	> 40	central and peripheral	A + C	none	none	congestion, follicle, papilla	mixed congestion	F+
10	redness, decrease in vision	21–40	central and peripheral	A + B	10 ~ 20	central and peripheral	congestion, follicle, papilla	congestion	F-
11	redness, watering, foreign-body sensation	> 40	central and peripheral	A + B+D	none	none	congestion, papilla	congestion	F-
12	redness,	> 40	central and peripheral	A	none	none	congestion, follicle, papilla	mixed congestion	F+
13	redness,	< 10	central	A	none	none	congestion, follicle	none	F-
14	redness,	< 10	peripheral	A + B	none	none	congestion, papilla	congestion	F+
15	redness	10 ~ 20	peripheral	D	10 ~ 20	peripheral	none	congestion	F+
16	redness, pain, photophobia, and watering, decrease in vision	< 10	central and peripheral	A + B	< 10	peripheral	congestion, follicle, papilla	mixed congestion, F+	F+
17	redness, itching, watering, decrease in vision	< 10	peripheral	A	none	none	congestion, follicle, papilla	congestion, F+	F+
18	redness, watering	< 10	central and peripheral	A + D	< 10	central and peripheral	congestion, follicle, papilla	congestion	F+
19	watering, decrease in vision	10 ~ 20	central and peripheral	C	< 10	central and peripheral	none	none	F-
20	watering, decrease in vision, foreign-body sensation	> 40	central and peripheral	A	< 10	central and peripheral	congestion, follicle	congestion	F+

*: (A) Small faint dots (B) Large (C) target and bizarre shaped (D) Thygeson’s like lesions; **F+: Fluorescein staining positive, F-: Fluorescein staining negative

**Table 3:** Previous diagnosis, treatment and prognosis of 20 Cases of MKC

Case NO.	previous diagnosis*	treatment**	prognosis	unilateral/bilateral
1	AK	0.02% PHMB	Cured, no recurrence	Left eye
2	DED, TSPK	0.02% PHMB, corneal epithelial curettages	Cured, no recurrence	Both eyes
3	TSPK	0.02% PHMB, fluorometholone, tacrolimus	Cured, no recurrence	Both eyes
4	DED, AKC	0.02% PHMB, 0.05% cyclosporine eye drops	Cured, no recurrence	Both eye
5	Vernal keratoconjunctivitis, TSPK, AKC	0.02% PHMB	Cured, no recurrence	Both eyes
6	AKC	0.02% PHMB	Cured, recurrence at 3 months, continued treatment and cured	Right eye
7	AC	0.02% PHMB, oral albendazole, 0.3% gatifloxacin eye drops	Cured, no recurrence	Right eye
8	AC	0.02% PHMB, oral albendazole, cyclosporin eye drops	Cured, no recurrence	Right eye
9	AKC	voriconazole	Cured, no recurrence	Both eyes
10	AKC	0.02% PHMB, oral albendazole	Cured, no recurrence	Right eye
11	AKC	0.02% PHMB	Cured, no recurrence	Both eyes
12	AKC	0.02% PHMB, oral albendazole	Cured, no recurrence	Right eye
13	DED, AKC	0.02% PHMB	Cured, no recurrence	Both eyes
14	AKC, AC	0.02% PHMB, oral albendazole	Cured, no recurrence	Right eye
15	NONE	refuse treatment	Spontaneously relieve, no recurrence	Left eye
16	AKC	0.02% PHMB, 0.05% cyclosporin eye drops	Cured, no recurrence	Both eyes
17	AKC	0.02% PHMB	Cured, no recurrence	Both eyes
18	AKC	0.02% PHMB	Cured, no recurrence	Both eyes
19	AKC	0.02% PHMB, oral albendazole	Cured, no recurrence	Both eyes
20	AKC	0.02% PHMB, 0.05% cyclosporin eye drops	Cured, no recurrence	Both eyes

* AK: acanthamoeba keratitis, DED: dry eye disease, TSPK: Thygeson superficial punctate keratitis, AKC: adenoviral keratoconjunctivitis, AC: allergic conjunctivitis, CEE: corneal epithelial exfoliation

**Fig. 3:**
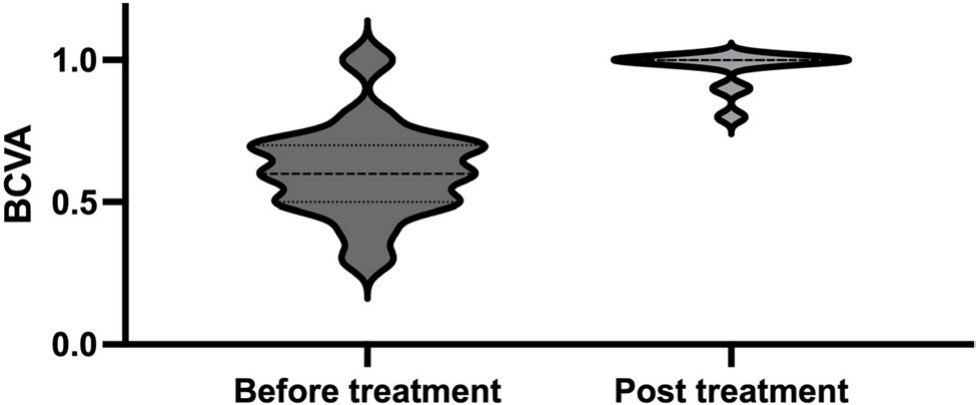
BCVA before and post treatment

Median BCVA before treatment was 0.6 (P25–P75: 0.5–0.7). Post-treatment median BCVA was 1.0 (P25–P75: 1.0–1.0). The Wilcoxon paired signed-rank test revealed a statistically significant difference, with *P* < 0.001. BCVA before and post treatment see Fig. 3.

Epithelial lesion grade vs. pre-treatment logMAR: Spearman’s *r* = 0.3686, *P* = 0.0379 (*P* < 0.05), significant weak positive correlation. Infiltration grade vs. post-treatment logMAR: Spearman’s *r* = 0.4088, *P* = 0.0202 (*P* < 0.05), significant weak-to-moderate positive correlation.

### Case 4

A 37-year-old female had worn daily disposable contact lenses for over 10 years. She presented with bilateral eye redness, pain, and decreased visual acuity for 2 months. Initially diagnosed with bilateral dry eyes or adenoviral keratoconjunctivitis, she received treatment with 0.1% fluorometholone, 0.05% cyclosporine, and deproteinized calf blood extract eye drops for a month without improvement. On ophthalmic examination, her best-corrected visual acuity was 20/25 in both eyes, accompanied by bilateral rough corneal epithelium and subepithelial infiltration. Further inquiry revealed that she had kept a hand-feeding parrot 3 months prior, and one month after feeding, she developed bilateral redness and pain sequentially. Three months after the onset of symptoms, bilateral corneal scrapings demonstrated microsporidia structures within epithelial cells, and bilateral metagenomic sequencing identified 314 sequences of *E. hellem*. Diagnosis: MKC (OU). After one month of topical 0.02% polyhexamethylene biguanide (PHMB) and 0.05% cyclosporine eye drops, her bilateral redness and pain significantly improved. These medications were continued for 3 months and then discontinued. At the end of treatment, her best-corrected visual acuity reached 20/20 in the right eye and 20/16 in the left eye. There was no conjunctival congestion, corneal opacity, or recurrence during the follow-up period.

### Case 6

A 45-year-old female tea artist had no obvious cause for intermittent redness and foreign-body sensation for 2 years, and the right eye was aggravated for 1 month after contacting outdoor spring water. She was previously treated as bilateral keratitis and dry eye for 5 months with relapse. Ophthalmic examination of the right eye showed conjunctival congestion, rough, elevated, spotted corneal and limbal epithelial lesions with positive fluorescein staining, and subepithelial corneal infiltration. The left eye showed superior bulbar conjunctival congestion with positive fluorescein staining. The microscopic examination of scraped corneal epithelial lesions reported spore-like structure. Metagenomic sequencing reported 34 sequences of *E. bieneusi.* Diagnosis: MKC (OD) and SLK (OS). After topical 0.02% PHMB treatment for 2.5 months, the conjunctival congestion of the right eye was relieved. Epithelial lesion and subepithelial infiltration were healed. After 3 months of treatment, the treatment was discontinued due to conscious improvement and Spring Festival holiday. Two weeks after discontinuation, rough, elevated, spotted corneal lesions reappeared. After second round of topical 0.02% PHMB treatment for 3 months, corneal lesions were healed and the medication was discontinued. No recurrence appeared during 10 months of follow-up.

### Case 7

A 13-year-old boy with 4-year orthokeratology lens wear had right eye redness and foreign body sensation for 2 weeks. He had prior diagnosis of allergic keratitis, unresponsive to anti-allergic treatment. His mother kept parrots for 6 months; one budgerigar developed “unilateral eye cold” 3 weeks prior, died a week later. His mother had high fever the next day, diagnosed with “psittacosis” and improved after treatment. Three days after the parrot’s onset, the boy had right eye redness and foreign body sensation, with eyelid swelling next morning.

Ophthalmic exam: right bulbar conjunctival hyperemia (Fig A), scattered punctate white elevated lesions on cornea and limbus, corneal fluorescein staining (CFS) (+); left eye normal. Corneal scraping showed spores; mNGS confirmed sequences of E. hellem infection. The boy was finally diagnosed as MKC (OD). Treated with 0.02% PHMB eye drops, 0.3% gatifloxacin eye drops for right eye, oral albendazole 400 mg twice daily, and parrot isolation. Three-month follow-up: no conjunctival hyperemia, clear cornea, CFS (-). No recurrence in 2 months after drug withdrawal.

### Case 16

A 15-year-old female with a 2-year history of OK Lens wear presented with 2-week left-eye redness, pain, photophobia, and watering, followed by 2-day right-eye redness. Antiviral and antibacterial therapies were ineffective. She denied ocular trauma or contaminated water exposure, but reported purchasing a pair of lovebird 1 month prior, which died 2 weeks before symptom onset. Ophthalmic examination revealed corrected visual acuity of 20/25 (right) and 20/200 (left). The right eye exhibited bulbar conjunctival congestion with scattered white corneal marginal infiltration; the left eye showed conjunctival congestion and central/inferior punctate elevated epithelial lesions. Microsporidia were identified in bilateral corneal scrapings, and bilateral mNGS confirmed 3 sequences of *E. hellem*. Diagnosis: MKC (OU). Treatment consisted of topical 0.02% PHMB and 0.05% cyclosporine. One week later, congestion improved, and corneal lesions diminished. At 1 month, only minimal punctate corneal limbal lesions persisted; by 3 months, cornea was clear with 20/20 corrected visual acuity bilaterally. Medication was discontinued, and no recurrence was observed during the 3-month follow-up.

### Case 17

A 43-year-old female, mother of Case 16, presented with bilateral eye redness, itching, watering and decrease in vision 2 weeks prior. Despite antiviral and antibacterial treatments, symptoms worsened. She denied ocular trauma or contaminated water exposure, but reported starting lovebird keeping 1 month before onset. Ophthalmic exam showed bilateral bulbar conjunctival congestion, corneal epithelial punctate elevations. Microsporidia were detected in bilateral corneal scrapings, and bilateral mNGS identified 3 sequences of *E. hellem*. Diagnosis: MKC (OU). Treatment included 0.02% PHMB qid (OU). Symptoms improved within a week. At 1-month follow-up, inferior corneal scattered punctate white lesions led to topical medication tapering to once daily for 3 months. No abnormalities or recurrence were noted during the 3-month post-withdrawal follow-up.

## Discussion

The spectrum of ocular infections from rare and emerging pathogens is expanding [15], warranting continuous clinical attention, as seen in the rising prevalence of ocular microsporidial infections in China [5]. Microsporidia, unicellular eukaryotic intracellular parasites, infect multiple human organs [1]. The eyes are the second - most common target of microsporidia, with the cornea being the primary site of infection [1]. As opportunistic pathogens, they affect both immunocompetent and immunocompromised individuals, causing microsporidial keratoconjunctivitis (MKC) or microsporidial stromal keratitis (MSK) [9]. Delayed diagnosis may lead to severe cases such as microsporidial endophthalmitis [16, 17]. In the past two decades, MKC cases in immunocompetent populations have increased, predominantly reported in India, Thailand, and Singapore [3, 4, 18]. Elsewhere, only case series or sporadic cases are documented [19–21]. MKC gained attention in mainland China only recently, after our first case report. This study, the largest MKC case series in Mainland China, aims to boost clinical awareness and understanding.

MKC caused by different species may vary in risk factors, transmission routes, clinical manifestations, and prognosis [1]. Therefore, accurate species identification is crucial. However, previous studies mainly focused on clinical manifestations and treatment outcomes. Although some studies have confirmed the causative species using PCR, only an Israeli study applied the more sensitive mNGS for species analysis [22]. Han et al. summarized 7 species belonging to 5 genera associated with MKC, including Anncaliia algerae, Encephalitozoon cuniculi, etc [1]. Among them, Encephalitozoon cuniculi and *E. hellem* are the most common [2]. *V. corneae* primarily causes MSK, but it has also been linked to MKC in the recent reports [2, 19]. Among 20 cases in this study, 13 had *E. hellem* infection, 3 had *V. corneae*, and 3 had *E. bieneusi*. Notably, *E. bieneusi* was reported for the first time as a causative agent of MKC. This parasite mainly infects the intestine and rarely causes systemic or respiratory diseases [1]. Its infection in immunocompetent patients was first linked to self-limiting chronic diarrhea in tropical travelers [23]. Previous studies showed *E. bieneusi* presence in surface water [24]. Case 6 developed MKC after 2 weeks of outdoor spring water exposure, indicating outdoor water exposure as a potential risk factor of *E. bieneusi*. Electron microscopy and PCR will further explore the association between *E. bieneusi* and ocular surface infections.

Identifying risk factors for MKC is essential for infection source tracing, disease prevention, and rapid clinical screening. Risk factors vary significantly across regions due to differences in economy, climate, and sanitation [2]. Reported risk factors of MKC included contaminated water exposure, soil/mud - related trauma, contact lens use, LASIK, immunosuppression, dust, insect bites, and animal contact [2]. Large - scale analyses from India and Singapore show contaminated soil, mud, or water exposure, especially during rain, is a major risk factor [4, 25]. However, in this case series, bird contact (1 pigeon, 14 psittacines, 75.0% of 20 cases) and contact lens use (3 orthokeratology, 1 soft lenses, 20.0% of 20 cases) are the primary risk factors.

This study reveals for the first time that contact with birds, especially psittacines, is a major risk factor for MKC, especially *E. hellem*-induced cases. Only two sporadic cases reported before, related to contacting Budgerigars or lovebirds [26, 27]. With the rising popularity of pet parrot ownership in China [28], the role of parrots as primary hosts of microsporidia has been overlooked. A total of 13 cases of *E. hellem*-induced MKC were detected in this study, all of them were clearly related to parrot keeping, with an onset 2–4 weeks after contact. Patients’ pet parrots included, but not limited to, lovebirds (majority), cockatoos, and eclectus. Ophthalmologists, particularly in emergency settings, should routinely inquire about bird (especially parrot) exposure when taking keratoconjunctivitis patients’ medical histories, aiming to reduce missed and misdiagnoses of MKC.

*E. hellem* is zoonotic and transmissible from parrots to humans. Psittacines are key reservoirs, infection sources and hosts of *E. hellem* [1]. Its spores are predominantly detected in avian feces [1]. Childs-Sanford et al. hypothesized that human infection of *E. hellem* occurred through contact with birds [29]. This study found that MKC might occur after contact with either healthy or diseased parrots, suggesting the zoonotic nature of *E. hellem*-induced MKC. Among patients’ parrots, diarrhea, ocular secretion, inter-parrot transmission, and parrot chick mortality were observed. *E. hellem* sequences were identified by conventional PCR and Sanger sequencing in fecal and conjunctival sac samples from the parrots of patient 4 (data not shown). These findings reinforce zoonotic potential of *E. hellem* and its parrot - to - human transmission risk, warranting further investigation. Notably, cases 16 and 17, a mother-daughter pair, developed MKC successively after close contact with lovebirds, indicating potential clustered cases of MKC caused by *E. hellem*. However, transmission routes remain elusive. Intriguingly, all 13 cases of parrot-keeping adopted hand-feeding rather than cage-feeding. Direct spore-hand-mucosa contact during hand-feeding might inoculate spores into the eyes, consistent with prior research [30]. Future studies will evaluate the homology of microsporidia between parrots and humans to strengthen evidence of zoonosis.

Contact lens use, especially orthokeratology (OK) lenses, is a risk factor for MKC and warrants clinical attention. Lenses can facilitate organism access to the cornea or promote colonization on their surfaces [31]. Most prior reports were sporadic cases, except for 25 cases reported by Loh et al. [4]. This study reports 3 MKC cases associated with OK - lens use, likely linked to the widespread use of OK lenses in mainland China. OK lenses may act as spore carriers [32]. Corneal epithelial barrier damage induced by orthokeratology may be an indirect cause of MKC, while exposure to microsporidia in the natural environment or carried by parrots may be the direct cause. Optometrists should closely monitor the lens care and the ocular health of lens wearers.

Laboratory diagnosis is crucial for confirmation of MKC. Transmission electron microscopy (TEM) is the gold standard, but it is usually difficult to obtain enough corneal and conjunctival epithelial tissue for TEM in clinical practice [2]. Therefore, corneal scraping and molecular tests are two primary diagnostic methods for etiological diagnosis of MKC, as extensively reported in the literature [3, 19, 33]. Appropriate staining methods enabled clear visualization of all stages of microsporidia. Various staining methods were used in MKC diagnosis. Joseph et al. found CFW staining was most effective (96.7%), followed by Modified Ziehl-Neelsen (93.3%), Gram (90%), and Giemsa (73.3%) [34]. Fan, Uematsu, and Agashe have used respectively Gram, Modified Ziehl-Neelsen, and CFW staining to diagnose multiple MKC cases [19, 35, 36]. Ghosh et al. suggested combining Chemifluorescent brighteners with traditional histological stains to improve diagnostic efficacy of MKC [37]. In this study, CFW and Giemsa staining were used in combination, capitalizing on their complementary advantages. CFW staining only detects mature spore walls (Fig. 2C and F), potentially yielding false - negatives for immature spores. In contrast, Giemsa staining shows both mature spores (unstained walls) [37] (Fig. 2B, E and G), and immature ones (meronts and sporonts, multinucleated) (Fig. 2E).

Redness, watering, irritation, lid swelling, foreign-body sensation, and variable decrease in vision were common and non-specific reported symptoms of keratoconjunctivitis, which included MKC [2]. This case series observed.

similar symptoms. Corneal signs: rough, multifocal, punctate, elevated round - oval epithelial lesions < 1 mm, with/without anterior stromal infiltration, was crucial for diagnosis. However, early-stage epithelial lesions were small, flat, and faintly punctate, indistinguishable from the punctate staining of dry eye. For example, case 4 was misdiagnosed as dry eye. Mohantya reported 4 various fluorescein staining pattern which represented different degree of epithelial lesion [2]. Intriguingly, fluorescein staining pattern might change after 3 days without symptoms in Cases 15 (Fig. 1K, L). Notably, positive fluorescein staining of limbus was might be a specific and common sign of MKC (seen in 13/20 cases), which should be further analyzed between various keratoconjunctivitis in future studies.

MKC should be clinically differentiated from TSPK, adenoviral keratoconjunctivitis (AKC), Acanthamoeba keratitis, atypical mycobacterial keratitis, dry eye disease, and epithelial keratitis of Herpes Simplex Virus in the vesicular stage [2, 9]. AKC and TSPK are two common misdiagnoses. This study has similar results. The multifocal, coarse, elevated plaque-like epithelial lesion with or without infiltration was a key distinguishing sign. Moreover, epithelial lesion may be counterstaining, and typical images are shown in Fig. 1G and L. This is consistent with the variable staining characteristics in the literature [2].

Currently, there are no standardized treatment guidelines for MKC. Ophthalmologists typically adopt diverse treatment approaches, guided by patients’ clinical responses. Previous studies have reported effective medications for MKC, including topical fluoroquinolones, 0.02% PHMB, 0.02% chlorhexidine, alone or with systemic albendazole [9]. Based on the efficacy of placebo treatment, one study proposed that MKC might be self-limiting [38]. However, in this study, most patients received treatment for 1 month or longer before confirmed diagnosis, and self-limiting trend was observed only in case 12. Treatment with topical 0.02% PHMB, with or without oral albendazole, and with or without topical 0.05% cyclosporine eye drops, led to improvement in 90.0% (18 out of 20) of patients, indicating that systemic anti-protozoal agents may not be necessary for immunocompetent individuals. Only Case 6 underwent recurrence 2 weeks after 3-month-treatment, which is similar to a former study [4]. This study also found poor BCVA before treatment correlated with more epithelial lesion, and poor BCVA post-treatment related to more stromal infiltration. Surgical intervention for MKC is rarely required. But for recurrent, unresponsive, or severe cases, early corneal debridement directly and rapidly removes the pathogens in the epithelium [35], such as in Case 2. However, corneal debridement may increase the risk of microsporidia penetrating into the corneal stroma, intensifying the patient’s pain, and secondary infection [9]. Therefore, it is generally not considered as the first-choice treatment.

## Conclusion

Conclusively, the small sample size is the main limitation of this case series. But as the first case series of Mainland China, this study identified *E. bieneusi* as a novel pathogenic species of MKC. Additionally, it reports, for the first time, MKC cases among OK lens wearers, as well as parrot-associated, family-clustering, and potentially zoonotic MKC cases—all of which were caused by *E. hellem* infections. This study yielded a misdiagnosis rate of 100% due to inadequate clinical awareness of MKC. Future research endeavors will recruit a larger patient cohort to detect the presence of *E. bieneusi*, validate the zoonotic nature of *E. hellem* through human-animal microsporidia homology analysis, and thereby furnish more robust evidence for the clinical prevention, diagnosis, and treatment of MKC.

## Data Availability

The datasets used of the current study are available from the corresponding author on reasonable request by email.

## Declarations

### Authors’ information

Dr Xu is associate Chief Physician, Deputy Director of Outpatient Department of The Affiliated Eye Hospital of Nanjing Medical University. Her research focuses on ocular pathogens, including the microsporidia, fungus and acanthamoeba, as well as contact - lens - related ocular surface problems.

## Abbreviations

CFS: Corneal fluorescein staining
CFW: Calcofluor white
MKC: Microsporidial keratoconjunctivitis
mNGS: Next-generation metagenomic sequencing
PHMB: Polyhexamethylene biguanide hydrochloride
TSPK: Thygeson superficial punctate keratitis
